# Supplementary material for: Real-time observation of Cooper pair splitting showing strong non-local correlations
Source: Nat Commun. 2021 Nov 4;12:6358. doi: 10.1038/s41467-021-26627-8 (PMC8569201; doi:10.1038/s41467-021-26627-8)
Supplement: Supplementary file 1 — Supplementary Information [file 41467_2021_26627_MOESM1_ESM.pdf]

Supplementary information:
Real-time observation of Cooper pair splitting showing strong non-local correlations

Antti Ranni,[1] Fredrik Brange,[2] Elsa T. Mannila,[2] Christian Flindt,[2] and Ville F. Maisi[1]

[1] *NanoLund and Solid State Physics, Lund University, Box 118, 22100 Lund, Sweden*

[2] *Department of Applied Physics, Aalto University, 00076 Aalto, Finland*

(Dated: October 6, 2021)

SUPPLEMENTARY NOTE 1: EXPERIMENTAL DETAILS

A. Island occupations and device parameter values

Strong Coulomb interactions allow us to control the occupation probabilities of the islands. For each island, the electrostatic energy reads $E_\alpha(n_\alpha) = E_{C\alpha}(n_\alpha - n_{G\alpha})^2$, where n_α is the number of excess electrons on the left or right island, $\alpha = L, R$. The charging energy is given as $E_{C\alpha} = e^2/2C_\alpha$ in terms of the total capacitance C_α of each island. We control the occupations via the parameters $n_{G\alpha} = C_{G\alpha}V_{G\alpha}/e$ by the gate voltages $V_{G\alpha}$ applied to the electrodes visible in Fig. 1**a** of the main text. By tuning the gate voltages so that $n_{G\alpha} = 1/2$, the charge states $n_\alpha = 0$ and $n_\alpha = 1$ become energetically degenerate with the corresponding charging energy diagram shown in Supplementary Fig. 1 below. This choice makes the Cooper pair splitting process occur at no energy cost. The charging energies of the islands arise predominantly from the self-capacitance of the 12 µm long islands [1], and we estimate the charging energies to be $E_{CL} = E_{CR} \sim 40\,\mu\text{eV}$ based on the values of Ref. 1. The tunnel junction transparency is estimated to be $170\,\text{k}\Omega\mu\text{m}^2$ based on the average transparency of three reference tunnel junctions fabricated during the same fabrication round with resistances 30 MΩ, 42 MΩ and 23 MΩ, and areas 75 nm × 68 nm, 80 nm × 80 nm and 63 nm × 63 nm correspondingly.

Coulomb interactions between the metallic islands would favor elastic cotunneling over Cooper pair splitting as the splitting process would require extra energy for occupying both islands with an electron. To avoid this, the device is designed so that the islands are as far from each other as possible, and each island is much closer to the gate lines and the ground plane than the other island. At the junctions, where the islands are closest, the grounded superconductor screens the island-to-island Coulomb interactions. In the measured time traces, we observe that the charge state of one island does not considerably affect the state of the other. Hence, we conclude that the inter-dot charging energy is negligible, and the total energy of the two islands is simply given by the individual contributions from each island.

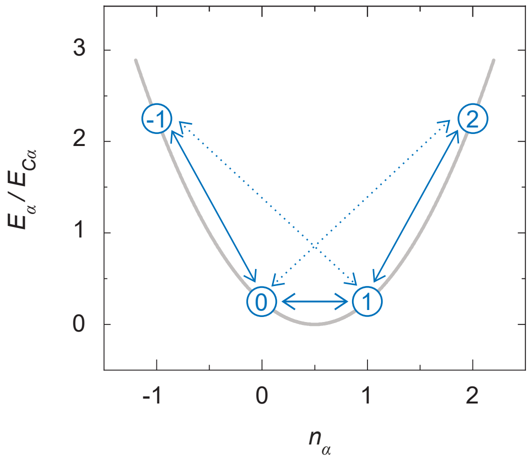

SUPPLEMENTARY FIGURE 1. **Charging energy diagram.** Lowest-lying charge states of island α, when the gate offset charge is tuned to $n_{g_\alpha} = 1/2$. With both islands at this degeneracy point, Cooper pair splitting is favorable, causing transitions from the charge state 0 to the charge state 1 in both islands.

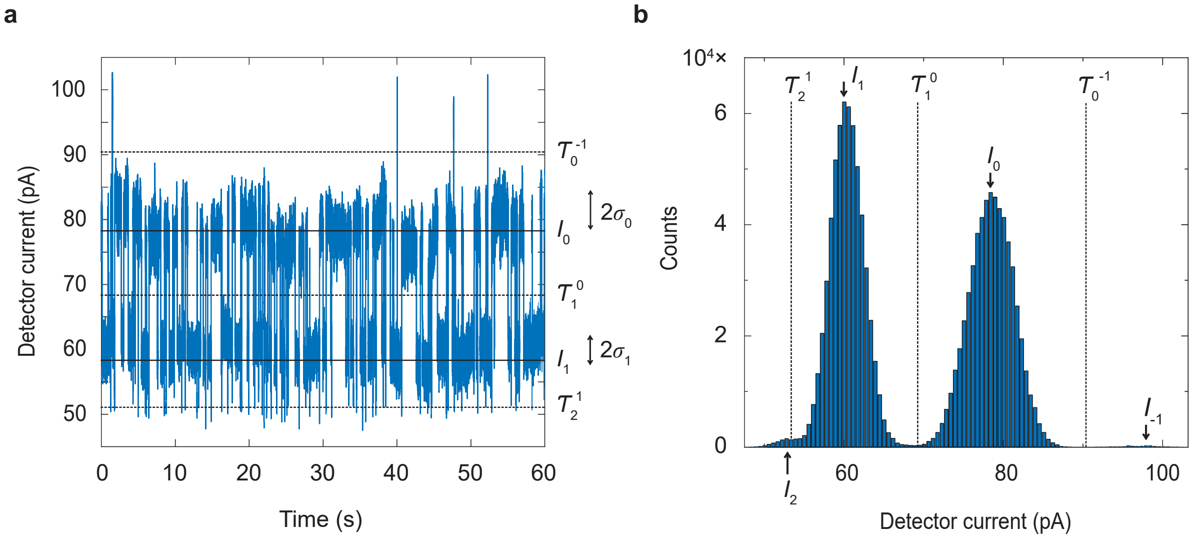

SUPPLEMENTARY FIGURE 2. **Identification of charge states.** **a,** Example of a time trace, where the thresholds T_j^i (dashed lines) divide the current according to the different charge states. The current levels, I_0 and I_1, of the lowest-lying charge states are indicated with solid black lines. **b,** Detector current from **a** divided into 100 bins. The peaks in the histogram correspond to the current levels of the four charge states of the each island. The current in each level j is given by a Gaussian distribution with standard deviation σ_j as indicated in panel **a**.

B. Identification of charge states and tunneling events

In the following, we explain how to determine the instantaneous charge states of the islands from the time traces. We also give the criteria by which some of the measured time traces were excluded from the analysis. The measured time traces, recorded at a sampling rate of 20 kHz simultaneously, were digitally filtered through a low-pass filter with a cut-off frequency of 200 Hz. This sets the detector rise times to $t_{\rm rise} = 4$ ms. 60 s long time traces were then analysed one by one. The detector current (see Supplementary Fig. 2**a**) is divided into a histogram (Supplementary Fig. 2**b**) consisting of 100 bins. The two largest peaks in the histogram are identified as the current levels of the two energetically lowest charge states, namely $n_\alpha = 0$ and $n_\alpha = 1$, corresponding to the most common states of the islands. The peaks are found by first locating the global maximum $I_{\rm h}$ in the current histogram and removing all values around it within $\pm 2\sigma$, where $\sigma = 2.5$ pA is the average standard deviation of the detector current at the charge states. From the remaining data in the histogram, the second maximum is located, and together these two maxima yield I_0 and I_1, such that I_0 is the state at the higher detector current, since an increasing number of electrons decreases the detector current.

The ratio N_0/N_1 of the number of counts N_i in the bins I_i yields the relative occupations of the states 0 and 1. If the offset charge n_{g_α} on island α differs considerably from the degeneracy point, $n_{g_\alpha} = 1/2$, the time spent in the charge states 0 and 1 are not equal. We maintain the system at the degeneracy value of $N_0/N_1 = 1$ by adjusting the gate voltages V_{g_α} in a feedback loop after the measurement of every time trace. To obtain the data from the main text, we use the time traces within the window $N_0/N_1 = 1/2$ to $N_0/N_1 = 2/1$.

Occasionally, the detector currents drift within the 60 s time trace such that one of the charge states drifts to the valley between I_0 and I_1, making it ambiguous to say whether an island is in the charge state 0 or 1. We discard these time traces using the following criteria: We examine how long time the detector current resides between I_0 and I_1 relative to the time spent at these two current levels by comparing N_0 and N_1 to the number of counts in between $N_{\rm valley}$. If there is a local maximum of the number of counts in the valley between the charge states 0 and 1 in the interval $[I_1 + 2\sigma, I_0 - 2\sigma]$ we take that as $N_{\rm valley}$. If there is no local maximum, we take the minimum in the same interval to be $N_{\rm valley}$. Then, the time trace is dismissed, if $N_{\rm valley}/\min(N_0, N_1) > 0.05$. With this condition, the charge states stay on the correct side of the threshold levels T_{i+1}^i of Supplementary Fig. 2**b** essentially at all times.

To determine the charge state of the islands, we set a threshold T_1^0 halfway between the peaks I_0 and I_1. Similarly,

if there is a local maximum in the histogram below $I_1 - 2\sigma$ (above $I_0 + 2\sigma$), we denote it as I_2 (I_{-1}) and set a threshold at the minimum between the peaks to distinguish the states 2 and -1. If we do not observe the maxima I_{-1} or I_2, we set no threshold and take all the data to be on state 0 or 1, correspondingly. Also, in order to avoid current noise induced false transitions, we have used a requirement that the detector current needs to get within $2\sigma_i$ of the charge state current I_i ($i = 0, 1$) to register an event between i and any of its neighbouring states. With the thresholds, we obtain the current ranges that allow us to determine the instantaneous charge state of the system and pinpoint the tunneling events as the points where at least one of the islands changes charge state.

C. Measurements of the correlation functions

The $g^{(2)}(\tau)$ correlation function describes how likely it is to observe an event at the time τ after another event took place. We determine the $g^{(2)}(\tau)$-functions in Fig. 1**b** and **c** of the main article directly based on the definition of the correlation function: We counted the number of events in a short time interval $\Delta\tau$ after time τ since the first event took place. The counting is straightforward after the identification of the tunneling events. The correlation function at time τ is determined then by normalizing the obtained counts with the appropriate normalization constant that depends on the total number of events, total measurement time and the width of the time interval as described in detail below. This normalization ensures the correct long time result of $g^{(2)}(\tau \to \pm\infty) = 1$. Importantly, the approach used here is the same for the auto-correlations and the cross-correlations with the only difference that in the auto-correlations, the two events are determined from the same detector and for the cross-correlations, the two events are from different detectors.

For the auto-correlation function $g^{(2)}(\tau)$ of Fig. 1**b** in the main text, we choose the counted events as follows: For each $0 \to 1$ event on the right island (the first event type), we count the number of $0 \to 1$ events taking place on the same island (the second event type) after a time τ around the time interval of $\Delta\tau = 100\,\mathrm{ms}$. This yields the correlation function $g^{(2)}(\tau)$ at the time separation τ, when divided by the normalization factor $(\dot{N}^R_{\mathrm{avg}})^2 \times t_{\mathrm{tot}} \times \Delta\tau$, where $\dot{N}^R_{\mathrm{avg}}$ is the total number of $0 \to 1$ events on the right island per total measurement time t_{tot}. The local one and two-electron tunneling processes were distinguished from each other before determining the correlation functions. The identification was made by taking two consecutive events happening within t_{rise} in the same detector to be local two-electron events and the rest to be sequential one-electron events. For example a local Andreev tunneling $-1 \to 1$ (consisting of closely happening consecutive $-1 \to 0$ and $0 \to 1$ transitions) was identified and did not contribute to $0 \to 1$ events in correlation functions.

In the auto-correlation measurements, the detector rise time t_{rise} limits the smallest τ that one may observe since the detector needs to respond before it can detect the next event. The time resolution of the auto-correlation measurement is visualized in Supplementary Fig. 3**a**, where we present a typical time trace yielding one count to the auto-correlation function. The count is obtained as two $0 \to 1$ events indicated with vertical dashed lines are observed in the right detector. The time τ indicates the time separation between the events and t_{rise} the detector rise time. This limitation is illustrated in Supplementary Fig. 4 that shows the auto-correlation function for sequential tunneling processes $0 \leftrightarrow 1$ on the right island. It is obtained similarly to the auto-correlation function in Fig. 1**b** of the main article with the only difference that here we account for both single-electron tunneling into and out of the island. An ideal detector with infinitely fast rise time would result in a flat $g^{(2)}(\tau)$ function because the considered tunneling events are uncorrelated, having the value one at each point in time even at $\tau = 0$ like the solid theory curve. Since our detectors have a finite rise time set by the bandwidth, we see a dip in the $g^{(2)}(\tau)$ function around zero time. The width of the dip matches t_{rise}, hence demonstrating the dead time of the detector limiting the short detection times for the auto-correlation function.

Returning now to the auto-correlation data of the $0 \to 1$ events presented in Fig. 1**b** of the main article, another timescale sets in: When detecting the two $0 \to 1$ events, the system needs to switch back to the initial state 0 before the second $0 \to 1$ event can be detected. Therefore the tunneling timescale to return from 1 to 0 yields a dip to the auto-correlation function. As seen in Fig. 1**b** of the main article, this dip is roughly one second wide. Thus, the much shorter t_{rise} does not have a considerable effect on our results on auto-correlation presented in the main text.

For the cross-correlation function $g_x^{(2)}(\tau)$ of Fig. 1**c** in the main text, we choose the counted events on separate islands. For the first event type we choose $0 \leftrightarrow 1$ transitions on the left island and count the number of $0 \leftrightarrow 1$

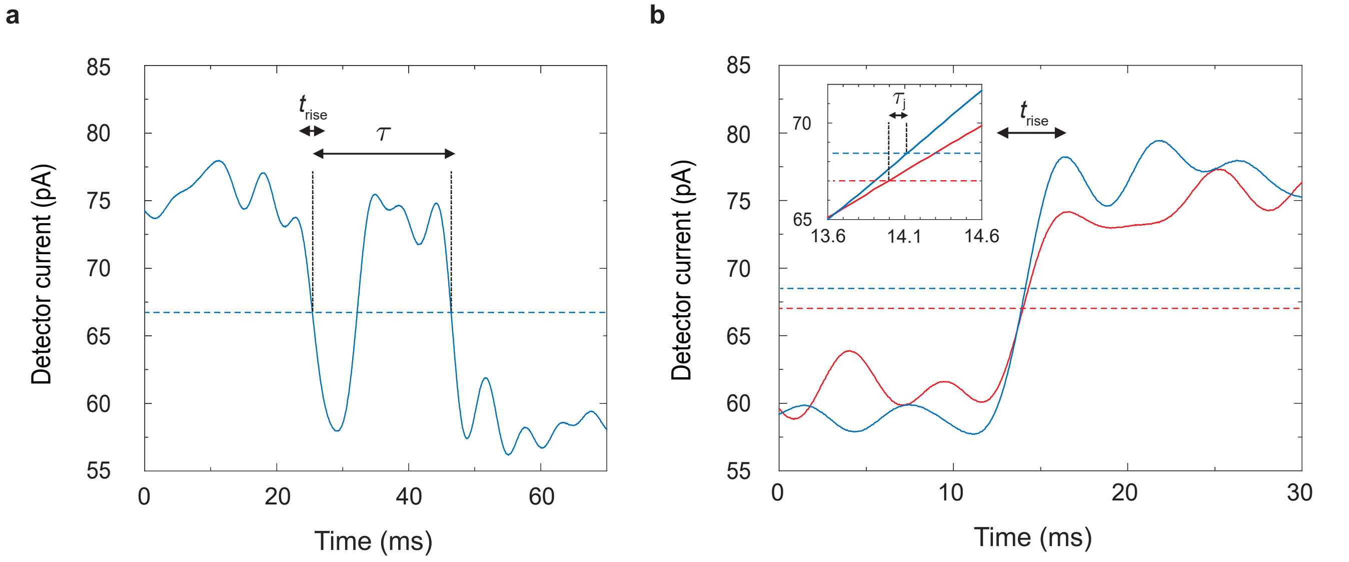

SUPPLEMENTARY FIGURE 3. **Time scales for detecting the tunneling events.** **a,** A typical time trace of the right detector yielding one count to the auto-correlation measurement. The solid line is the measured detector signal and the horizontal dashed line indicates the threshold between charge states 0 (above the line) and 1 (below). The time τ is the separation between two $0 \to 1$ transitions. The detector rise time t_{rise} sets a limit to how quickly the two events can be observed with the same detector. **b,** A typical time trace yielding one count to the cross-correlation measurement. Detector currents for the left and the right islands are shown in red and blue curves respectively. The detectors switch almost simultaneously from the charge state 1 to 0. The observed time separation $\tau_{\mathrm{j}} \ll t_{\mathrm{rise}}$ between the $1 \to 0$ events on the two distinct islands is indicated in the zoom-in of the inset.

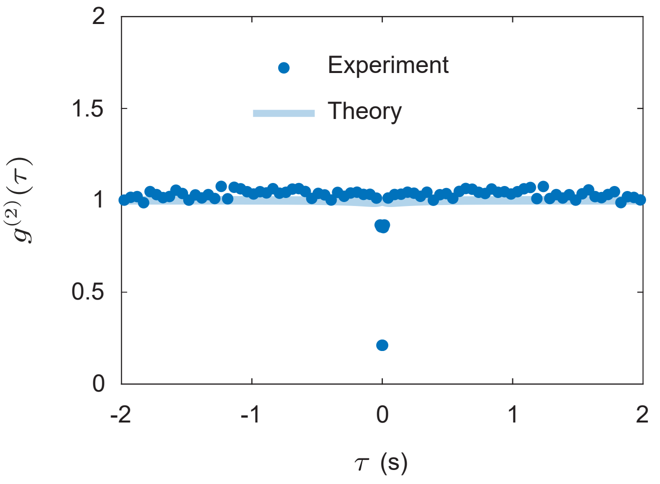

SUPPLEMENTARY FIGURE 4. **Influence of the finite detector rise time to correlation measurements.** Auto-correlation function for $0 \to 1$ and $1 \to 0$ processes on the right island. The experimental points close to zero time show a millisecond-wide dip due to the finite rise time of the detector.

transitions on the right island after a time τ around the short time interval of $\Delta\tau = 150\,\mu\mathrm{s}$. Detector currents are recorded every $50\,\mu\mathrm{s}$ and the interval is chosen commensurate to this such that each interval contains three sampling points. This is achieved by centering one interval $\Delta\tau$ at $\tau = 0$ and adding intervals next to each other towards positive and negative τ values. The normalization factor for the cross-correlation is $\dot{N}^L_{\mathrm{avg}} \times \dot{N}^R_{\mathrm{avg}} \times t_{\mathrm{tot}} \times \Delta\tau$ as we now account for the total number of events $\dot{N}^{\alpha}_{\mathrm{avg}}$ between charge states 0 and 1 on both islands α per total measurement time t_{tot}.

Supplementary Figure 3**b** presents the timing resolution of the cross-correlation measurements. The measured time

traces exhibit nearly simultaneously switching that yields one count to the cross-correlation function of Fig. 1**c** of the main article. The time difference τ of the splitting events arises from the detector timing jitter $\tau_{\rm j}$ presented in the inset. Interestingly, the restriction on the time resolution from the finite rise time $t_{\rm rise}$ is lifted when monitoring events with two distinct detectors. When having an own detector for both tunneling events, neither of the detectors needs to recover from detection of the first event and hence the detector dead time is not relevant. We observe this directly in the measured time traces of Supplementary Fig. 3**b**. The observed time separation $\tau_{\rm j}$ between the two transitions is much smaller than the rise time $t_{\rm rise}$ of the individual detectors. This finding is valid for all the events at the correlation peak of Fig. 1**c** of the main article. Hence, we demonstrate a sub-millisecond time resolution despite the detector rise time is in the millisecond range.

The relevant limiting factor for the time resolution of the cross-correlation measurement arises from the detector noise. Noise in the measured electrical current translates into noise in the timing of the detectors [2]. This is known as the timing jitter that we denoted above with $\tau_{\rm j}$. The relation of the current noise σ_{I_i} and the timing jitter (i.e. the 'time noise') $\sigma_{\tau_{\rm j}}$ is given by the slew rate dI/dt of the detector. The slew rate characterizes how quickly the detector responds to a change and is directly obtained from the derivatives of the detector response of Supplementary Fig. 3**b** near the thresholds. Similarly the current noise σ_{I_i} is obtained from the standard deviation of the current within the charge state i, cf. Supplementary Fig. 2. With these, we obtain $\sigma_{\tau_{\rm j}(i\to f)} = |dI/dt|^{-1}\sigma_{I_i}$. The slew rates, obtained directly from the traces, are 6.7 pA/ms and 8.4 pA/ms for $0 \to 1$ and $1 \to 0$ transitions on the right island respectively. On the left island the slew rate is 5.4 pA/ms to both directions. The current noises for the left detector are $\sigma_{I_0} = 2.2$ pA, $\sigma_{I_1} = 1.7$ pA, and for the right detector $\sigma_{I_0} = 2.8$ pA and $\sigma_{I_1} = 2.2$ pA. The slew rates together with the current noises yield the detector timing jitters $\sigma_{\tau_{\rm j}(0\to1)} = 410$ μs, $\sigma_{\tau_{\rm j}(1\to0)} = 310$ μs for the left detector and $\sigma_{\tau_{\rm j}(0\to1)} = 420$ μs and $\sigma_{\tau_{\rm j}(1\to0)} = 260$ μs for the right detector. Then the detector broadening of the $g_x^{(2)}(\tau)$ function is determined as weighted sums of each participating charge state, see Equation (9). The timing jitters together with the tunneling rates below are all the input parameters needed for the theory curves without any further fitting.

D. Tunneling rates

To identify which tunneling process took place with each event we followed the procedure of Ref. 1: We interpret all events that take place within the detector rise time of 4 ms at the same island to belong to the same event. Supplementary Figure 5 presents typical cases taking place around the charge state $n_R = 0$ of the right island. Panel a summarizes the life-time distribution of the charge state if all events at the detector would be treated as sequential single-electron events. In this case, the bin at the shortest time within the detector rise time have anomalously many events whereas all the rest of the data follows an exponential distribution as expected for tunneling starting from $n_R = 0$. The reason for the anomalously high first data point is that those events belong to a process that started from another charge state and should not be counted into $n_R = 0$ state [1]. Panel b shows an example time trace with such events.

In Supplementary Fig. 5**b** we see in the beginning of the time trace a transition $n_R = 1 \to 0$ followed immediately within the detector rise time by $n_R = 0 \to -1$ yielding us a two electron local Andreev tunneling event. The other case contributing to short time counts of panel a is also shown in Supplementary Fig. 5**b**. In this case the systems seems to take transition $1 \to 0$ followed quickly by another event with $0 \to 1$. Two such cases are visible in the end of the time trace. However, based on the lifetime distribution, there are too many of the latter return transitions $0 \to 1$ for them to arise from the $0 \to 1$ single-electron transition. Therefore, we interpret this course of events to arise from two local Andreev transitions, one with $n_R = 1 \to -1$ followed by $n_R = -1 \to 1$. The second transition takes place quickly after the first one as it has an energy gain, see the energy diagram of Supplementary Fig. 1. The tunneling rate for the return process is so fast that the detector does not have time to reach the final state before starting to return to the initial state. Thus the $1 \leftrightarrow -1$ transition appears as if it would be between $1 \leftrightarrow 0$. Due to this ambiguity, we exclude the cases with a fast return event (lifetime at state 0 shorter than 4 ms) from the determination of the tunneling rates. We note that these events take place locally on one of the islands and hence they do not impact the results presented in the main article. If we take them into the analysis as Andreev events the correlation function results remain unchanged and the hold time would still remain two orders of magnitude longer than the detection time.

With the above protocol we distinguish different local tunneling processes. In addition, we identify the crossed processes as those $0 \leftrightarrow 1$ events that take place on different islands within the 1.5 ms time window obtained from the cross-correlation function and finally determine the tunneling rates by counting how many times the corresponding

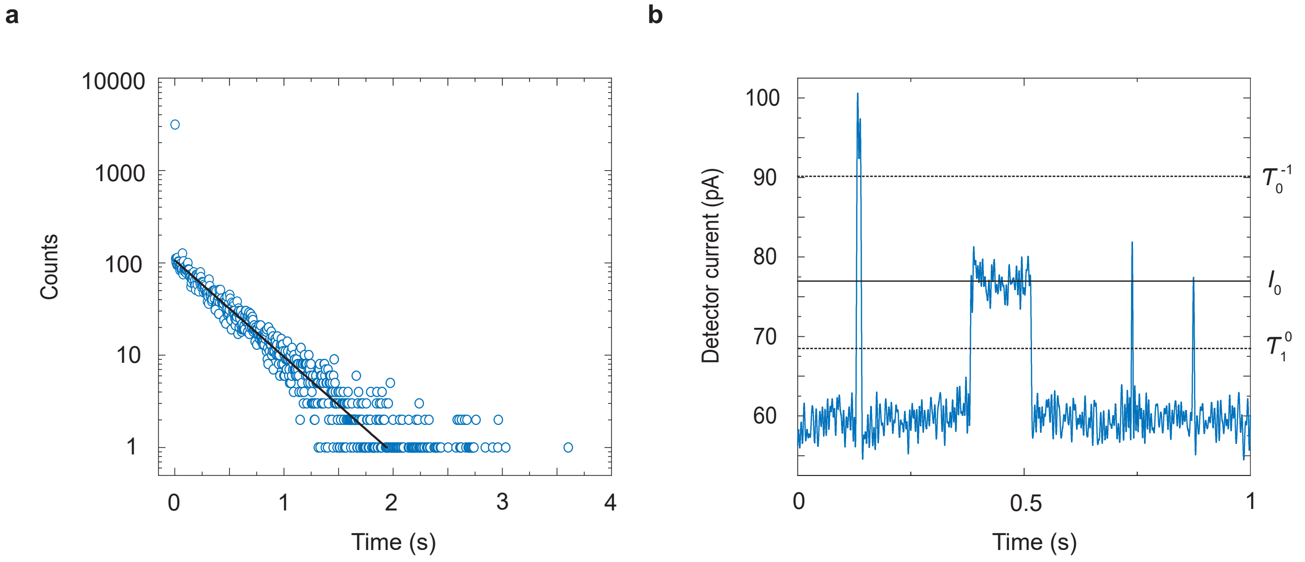

SUPPLEMENTARY FIGURE 5. **Life-time distribution. a,** Time spent in the charge state 0 of the right island before a tunneling event out of it. The bin size is 4 ms, and the first point arises due to Andreev tunneling that started from another state. The long-time statistics follow an exponential distribution which determines the life-time of the state. **b,** The leftmost peak shows a local Andreev tunneling from $n_R = 1 \rightarrow -1$ and its time reversal process to opposite direction. Duration the detector current spends at $n_R = 0$ is less than the detector rise time of 4 ms. The wide peak in the middle of the graph corresponds to a sequential tunneling $n_R = 1 \rightarrow 0$ and its time reversal process. The two sharp peaks on the right side are local Andreev tunneling events which take the right island $n_R = 1 \rightarrow -1$ and are followed by another local Andreev back to $n_R = 1$ but these events happen in a time shorter than the detector rise time and thus it appears as if there were tunneling sequences $n_R = 1 \rightarrow 0 \rightarrow 1$.

SUPPLEMENTARY TABLE I. Tunneling rates for the processes on individual islands (L for the left island and R for the right one).

	$\Gamma_{1\to0}$ (Hz)	$\Gamma_{0\to1}$ (Hz)	$\Gamma_{2\to1}$ (Hz)	$\Gamma_{1\to2}$ (Hz)	$\Gamma_{0\to-1}$ (Hz)	$\Gamma_{-1\to0}$ (Hz)	$\Gamma_{2\to0}$ (Hz)	$\Gamma_{0\to2}$ (Hz)	$\Gamma_{1\to-1}$ (Hz)	$\Gamma_{-1\to1}$ (Hz)
L	0.11	0.084	700	0.017	0.21	7.2	160	0.0026	0.11	3.8
R	1.6	2.3	9.2	0.69	0.024	4.4	26	0.0080	0.32	29

tunneling process occurred and divide with the time spent in the initial state. The tunneling rates for local processes are listed in Supplementary Table I. The non-local crossed tunneling rates are $\Gamma^{\mathrm{R}=0\to1}_{\mathrm{L}=0\to1} = 14$ mHz for Cooper pair splitting, $\Gamma^{\mathrm{R}=1\to0}_{\mathrm{L}=1\to0} = 140$ mHz for Cooper pair assembling, $\Gamma^{\mathrm{R}=0\to1}_{\mathrm{L}=1\to0} = 25$ mHz for elastic cotunneling from the left island into the right one, and $\Gamma^{\mathrm{R}=1\to0}_{\mathrm{L}=0\to1} = 96$ mHz for elastic cotunneling from the right island into the left one.

E. Waiting time distributions

The waiting time distribution in Fig. 3**a** of the main text is obtained by determining the time duration τ from a $1 \rightarrow 0$ transition to the next $1 \rightarrow 0$ transition locally on the right detector. These transitions are either sequential tunneling events solely on the right island or arise from crossed processes moving the right system from $n_R = 1$ to $n_R = 0$. Between two $1 \rightarrow 0$ transitions, any other type of tunneling process is allowed to take place. For example, after a $1 \rightarrow 0$ transition we can have a sequence $0 \rightarrow 1 \rightarrow -1 \rightarrow 1$ before a single electron tunnels out again from the state 1. Hence, the waiting time describes the time scale one needs to wait until the same event occurs again. To obtain the distribution, we again count the number transitions taking a time τ since the previous transition around a short time interval of $\Delta\tau$. This yields the waiting time distribution $\mathcal{W}(\tau)$, when divided by the normalization factor $N \times \Delta\tau$ where N is the total number of counts to all bins.

For the cross-waiting time distribution in Fig. 3**b** of the main text, we consider the simultaneously measured time traces on the two islands and find the waiting times τ from each $1 \rightarrow 0$ transition on the left island to the consecutive $1 \rightarrow 0$ event on the right island. During the waiting time τ any tunneling process on the left island is allowed, even

another $1 \to 0$ transition. The cross-waiting time distribution $\mathcal{W}_x(\tau)$ is computed the same way as $\mathcal{W}(\tau)$.

SUPPLEMENTARY NOTE 2: THEORETICAL MODEL

In the following, we develop a theoretical model of the Cooper pair split, which we use to derive all of the theoretical results in the main text. To this end, we employ a rate equation to describe the dynamics of the two islands,

$$\frac{d}{dt}|p(t)\rangle\!\rangle = \mathbf{L}|p(t)\rangle\!\rangle, \tag{1}$$

where $|p(t)\rangle\!\rangle = (p_1 \quad p_2 \quad ... \quad p_{16})^T$ is a column vector containing the probabilities p_i of being in the 16 different charge states of the islands, and $\mathbf{L}$ is a rate matrix. $\mathbf{L}$ has the off-diagonal elements $\mathbf{L}_{ij} = \Gamma_{j\to i}$, $i \neq j$, and the diagonal elements $\mathbf{L}_{jj} = -\sum_{i\neq j}\Gamma_{j\to i}$, with $\Gamma_{j\to i}$ being the transition rate from the jth to the ith state. To be explicit, the off-diagonal part of $\mathbf{L}$ reads

$$\begin{pmatrix}
0 & \Gamma^R_{0\to-1} & \Gamma^R_{1\to-1} & 0 & \Gamma^L_{0\to-1} & 0 & 0 & 0 & \Gamma^L_{1\to-1} & 0 & 0 & 0 & 0 & 0 & 0 & 0 \\
\Gamma^R_{-1\to0} & 0 & \Gamma^R_{1\to0} & \Gamma^R_{2\to0} & 0 & \Gamma^L_{0\to-1} & 0 & 0 & 0 & \Gamma^L_{1\to-1} & 0 & 0 & 0 & 0 & 0 & 0 \\
\Gamma^R_{-1\to1} & \Gamma^R_{0\to1} & 0 & \Gamma^R_{2\to1} & 0 & 0 & \Gamma^L_{0\to-1} & 0 & 0 & 0 & \Gamma^L_{1\to-1} & 0 & 0 & 0 & 0 & 0 \\
0 & \Gamma^R_{0\to2} & \Gamma^R_{1\to2} & 0 & 0 & 0 & 0 & \Gamma^L_{0\to-1} & 0 & 0 & 0 & \Gamma^L_{1\to-1} & 0 & 0 & 0 & 0 \\
\Gamma^L_{-1\to0} & 0 & 0 & 0 & 0 & \Gamma^R_{0\to-1} & \Gamma^R_{1\to-1} & 0 & \Gamma^L_{1\to0} & 0 & 0 & 0 & \Gamma^L_{2\to0} & 0 & 0 & 0 \\
0 & \Gamma^L_{-1\to0} & 0 & 0 & \Gamma^R_{-1\to0} & 0 & \Gamma^R_{1\to0} & \Gamma^R_{2\to0} & 0 & \Gamma^L_{1\to0} & \Gamma^{\mathrm{CAR}}_{\mathrm{in}} & 0 & 0 & \Gamma^L_{2\to0} & 0 & 0 \\
0 & 0 & \Gamma^L_{-1\to0} & 0 & \Gamma^R_{-1\to1} & \Gamma^R_{0\to1} & 0 & \Gamma^R_{2\to1} & 0 & \Gamma^{\mathrm{EC}}_{L\to R} & \Gamma^L_{1\to0} & 0 & 0 & 0 & \Gamma^L_{2\to0} & 0 \\
0 & 0 & 0 & \Gamma^L_{-1\to0} & 0 & \Gamma^R_{0\to2} & \Gamma^R_{1\to2} & 0 & 0 & 0 & 0 & \Gamma^L_{1\to0} & 0 & 0 & 0 & \Gamma^L_{2\to0} \\
\Gamma^L_{-1\to1} & 0 & 0 & 0 & \Gamma^L_{0\to1} & 0 & 0 & 0 & 0 & \Gamma^R_{0\to-1} & \Gamma^R_{1\to-1} & 0 & \Gamma^L_{2\to1} & 0 & 0 & 0 \\
0 & \Gamma^L_{-1\to1} & 0 & 0 & 0 & \Gamma^L_{0\to1} & \Gamma^{\mathrm{EC}}_{R\to L} & 0 & \Gamma^R_{-1\to0} & 0 & \Gamma^R_{1\to0} & \Gamma^R_{2\to0} & 0 & \Gamma^L_{2\to1} & 0 & 0 \\
0 & 0 & \Gamma^L_{-1\to1} & 0 & 0 & \Gamma^{\mathrm{CAR}}_{\mathrm{out}} & \Gamma^L_{0\to1} & 0 & \Gamma^R_{-1\to1} & \Gamma^R_{0\to1} & 0 & \Gamma^R_{2\to1} & 0 & 0 & \Gamma^L_{2\to1} & 0 \\
0 & 0 & 0 & \Gamma^L_{-1\to1} & 0 & 0 & 0 & \Gamma^L_{0\to1} & 0 & \Gamma^R_{0\to2} & \Gamma^R_{1\to2} & 0 & 0 & 0 & 0 & \Gamma^L_{2\to1} \\
0 & 0 & 0 & 0 & \Gamma^L_{0\to2} & 0 & 0 & 0 & \Gamma^L_{1\to2} & 0 & 0 & 0 & 0 & \Gamma^R_{0\to-1} & \Gamma^R_{1\to-1} & 0 \\
0 & 0 & 0 & 0 & 0 & \Gamma^L_{0\to2} & 0 & 0 & 0 & \Gamma^L_{1\to2} & 0 & 0 & \Gamma^R_{-1\to0} & 0 & \Gamma^R_{1\to0} & \Gamma^R_{2\to0} \\
0 & 0 & 0 & 0 & 0 & 0 & \Gamma^L_{0\to2} & 0 & 0 & 0 & \Gamma^L_{1\to2} & 0 & \Gamma^R_{-1\to1} & \Gamma^R_{0\to1} & 0 & \Gamma^R_{2\to1} \\
0 & 0 & 0 & 0 & 0 & 0 & 0 & \Gamma^L_{0\to2} & 0 & 0 & 0 & \Gamma^L_{1\to2} & 0 & \Gamma^R_{0\to2} & \Gamma^R_{1\to2} & 0
\end{pmatrix}.$$

Together with the diagonal part, each column of the rate matrix sums to zero, ensuring that the total probability of being in any of the states is preserved over time. The jump operator $\mathbf{J}_\alpha$ of a certain kind of transition α contains all off-diagonal matrix elements of $\mathbf{L}$ corresponding to the processes involving that transition. We note that the crossed Andreev reflections enter here as one of the possible processes. For instance, the jump operator $\mathbf{J}^L_{1\to0}$ contains all off-diagonal matrix elements of $\mathbf{L}$ that involve the transition $1 \to 0$ on the left island, including crossed Andreev and elastic cotunneling processes.

A. Second-order correlation functions

We define the $g^{(2)}$-correlation function of two types of transitions α and β as [3]

$$g^{(2)}(t, t+\tau) = \frac{P_{\beta\alpha}(t+\tau, t)}{P_\beta(t+\tau)P_\alpha(t)}, \tag{2}$$

where $P_\alpha(t)$ is the probability density of transition α taking place at time t, and $P_{\beta\alpha}(t+\tau, t)$ is the joint probability density of transition α taking place at t and transition β at $t+\tau$. For a steady state, the $g^{(2)}$-function depends only on the time difference between the transitions, $g^{(2)}(t, t+\tau) \equiv g^{(2)}(\tau)$. In that case, we may express Eq. (2) in terms of jump operators as [4]

$$g^{(2)}(\tau) = \frac{\langle\!\langle \mathbf{J}_\beta e^{\mathbf{L}\tau}\mathbf{J}_\alpha\rangle\!\rangle}{\langle\!\langle \mathbf{J}_\beta\rangle\!\rangle\langle\!\langle \mathbf{J}_\alpha\rangle\!\rangle}\theta(\tau) + \frac{\langle\!\langle \mathbf{J}_\alpha e^{-\mathbf{L}\tau}\mathbf{J}_\beta\rangle\!\rangle}{\langle\!\langle \mathbf{J}_\beta\rangle\!\rangle\langle\!\langle \mathbf{J}_\alpha\rangle\!\rangle}\theta(-\tau) + \frac{\langle\!\langle \mathbf{J}_{\alpha\beta}\rangle\!\rangle}{\langle\!\langle \mathbf{J}_\beta\rangle\!\rangle\langle\!\langle \mathbf{J}_\alpha\rangle\!\rangle}\delta(\tau), \tag{3}$$

where $\langle\!\langle \mathbf{A}\rangle\!\rangle \equiv \langle\!\langle \tilde{0}|\mathbf{A}|p_s(t)\rangle\!\rangle$ is the expectation value with respect to the steady state fulfilling $\mathbf{L}|p_s(t)\rangle\!\rangle = 0$, $\langle\!\langle \tilde{0}|$ is the vector representation of the trace operation, $\theta(\tau)$ is the Heaviside step function, and $\delta(\tau)$ is the Dirac delta function.

The last term in Equation (3) accounts for instantaneous correlations between electrons belonging to the same two-electron process. Such processes are described by two-electron superoperators $\mathbf{J}_{\alpha\beta} = (\mathbf{J}_\alpha \circ \mathbf{J}_\beta)^{\circ 1/2}(1-\delta_{\alpha\beta})$, with $\circ$ denoting Hadamard operations (element-wise operations) and $\delta_{\alpha\beta}$ the Kronecker delta function, containing all the matrix elements involving both transitions α and β. We note here that the instantaneous correlations are strongly affected by the timing jitter of the detectors, which effectively broadens the delta spike in time. The timing jitter is accounted for by convolving the correlation function with a Gaussian distribution of width σ_D, as discussed below for the cross-correlation $g_x^{(2)}(\tau)$-function.

1. *Equation (1) in the main text*

We first consider the auto-correlation $g^{(2)}$-function for electrons tunneling out of the right island into the superconductor. To this end, we set $\mathbf{J}_\alpha = \mathbf{J}_\beta = \mathbf{J}^R_{1\to 0}$ and $\mathbf{J}_{\alpha\beta} = 0$ in Equation (3), yielding the expression

$$g^{(2)}(\tau) = \frac{\langle\langle \mathbf{J}^R_{1\to 0} e^{\mathbf{L}|\tau|} \mathbf{J}^R_{1\to 0}\rangle\rangle}{\langle\langle \mathbf{J}^R_{1\to 0}\rangle\rangle^2}. \tag{4}$$

Next, we make use of the fact that the two-electron processes involving also the left island are slow compared to the dynamics of the right island. To compute the auto-correlation $g^{(2)}$-function, we may therefore treat the right island as an approximately separate four-level system, with the rate matrix

$$\mathbf{L}_R = \begin{pmatrix} -(\Gamma^R_{\text{-}1\to 0} + \Gamma^R_{\text{-}1\to 1}) & \Gamma^R_{0\to\text{-}1} & \Gamma^R_{1\to\text{-}1} & 0 \\ \Gamma^R_{\text{-}1\to 0} & -(\Gamma^R_{0\to\text{-}1} + \Gamma^R_{0\to 1} + \Gamma^R_{0\to 2}) & \Gamma^R_{1\to 0} & \Gamma^R_{2\to 0} \\ \Gamma^R_{\text{-}1\to 1} & \Gamma^R_{0\to 1} & -(\Gamma^R_{1\to\text{-}1} + \Gamma^R_{1\to 0} + \Gamma^R_{1\to 2}) & \Gamma^R_{2\to 1} \\ 0 & \Gamma^R_{0\to 2} & \Gamma^R_{1\to 2} & -(\Gamma^R_{2\to 0} + \Gamma^R_{2\to 1}) \end{pmatrix}. \tag{5}$$

With the total rates out of the states -1 and $+2$ being much larger than all other rates, we find that the right island effectively behaves as a two-level system with the high-energy states -1 and $+2$ as short-lived virtual states, resulting in the correlation function

$$g^{(2)}(\tau) = 1 - \exp\left[-\left(\widetilde{\Gamma}^R_{0\to 1} + \widetilde{\Gamma}^R_{1\to 0}\right)|\tau|\right], \tag{6}$$

which is the expected functional form of the $g^{(2)}$-function of a two-level system. However, here $\widetilde{\Gamma}^R_{0\to 1}$ and $\widetilde{\Gamma}^R_{1\to 0}$ are the *effective* transition rates between the two levels, which to leading order in the ratio between the total rates out of the virtual states and the other transition rates are

$$\begin{aligned} \widetilde{\Gamma}^R_{0\to 1} &= \Gamma^R_{0\to 1} + \frac{\Gamma^R_{0\to -1}\Gamma^R_{-1\to 1}}{\Gamma^R_{-1\to 0} + \Gamma^R_{-1\to 1}} + \frac{\Gamma^R_{0\to 2}\Gamma^R_{2\to 1}}{\Gamma^R_{2\to 0} + \Gamma^R_{2\to 1}} = 2.3\ \text{s}^{-1}, \\ \widetilde{\Gamma}^R_{1\to 0} &= \Gamma^R_{1\to 0} + \frac{\Gamma^R_{1\to -1}\Gamma^R_{-1\to 0}}{\Gamma^R_{-1\to 0} + \Gamma^R_{-1\to 1}} + \frac{\Gamma^R_{1\to 2}\Gamma^R_{2\to 0}}{\Gamma^R_{2\to 0} + \Gamma^R_{2\to 1}} = 2.2\ \text{s}^{-1}, \end{aligned} \tag{7}$$

where the last two terms in each equation describe the contributions from the virtual states in addition to the bare rates $\Gamma^R_{0\to 1}$ and $\Gamma^R_{1\to 0}$. Introducing $\gamma \equiv \widetilde{\Gamma}^R_{0\to 1} + \widetilde{\Gamma}^R_{1\to 0} = 4.5\ \text{s}^{-1}$, we recover Equation (1) of the main text from Equation (6). An exact numerical calculation based on Equation (4) shows good agreement with the analytic result in Equation (6), although the exact numerical value of γ is slightly higher due to the contributions from the two-particle processes involving both islands that are omitted in the approximations above.

2. *Equation (2) in the main text*

Next, we consider the cross-correlation $g^{(2)}$-function between electrons tunneling into or out of the left island and electrons tunneling into or out of the right island. In this case, we have $\mathbf{J}_\alpha = \mathbf{J}^L_{0\to 1} + \mathbf{J}^L_{1\to 0}$, $\mathbf{J}_\beta = \mathbf{J}^R_{0\to 1} + \mathbf{J}^R_{1\to 0}$ and $\mathbf{J}_{\alpha\beta} = \mathbf{J}^{\text{CAR}}_{\text{in}} + \mathbf{J}^{\text{CAR}}_{\text{out}} + \mathbf{J}^{\text{EC}}_{L\to R} + \mathbf{J}^{\text{EC}}_{R\to L}$ in Equation (3) and obtain

$$\begin{aligned} g_x^{(2)}(\tau) = &\frac{\langle\langle(\mathbf{J}^R_{0\to 1} + \mathbf{J}^R_{1\to 0})e^{\mathbf{L}\tau}(\mathbf{J}^L_{0\to 1} + \mathbf{J}^L_{1\to 0})\rangle\rangle}{\langle\langle\mathbf{J}^R_{0\to 1} + \mathbf{J}^R_{1\to 0}\rangle\rangle\langle\langle\mathbf{J}^L_{0\to 1} + \mathbf{J}^L_{1\to 0}\rangle\rangle}\theta(\tau) + \frac{\langle\langle(\mathbf{J}^L_{0\to 1} + \mathbf{J}^L_{1\to 0})e^{-\mathbf{L}\tau}(\mathbf{J}^R_{0\to 1} + \mathbf{J}^R_{1\to 0})\rangle\rangle}{\langle\langle\mathbf{J}^R_{0\to 1} + \mathbf{J}^R_{1\to 0}\rangle\rangle\langle\langle\mathbf{J}^L_{0\to 1} + \mathbf{J}^L_{1\to 0}\rangle\rangle}\theta(-\tau) \\ &+ \frac{\langle\langle\mathbf{J}^{\text{CAR}}_{\text{in}}\rangle\rangle + \langle\langle\mathbf{J}^{\text{CAR}}_{\text{out}}\rangle\rangle + \langle\langle\mathbf{J}^{\text{EC}}_{L\to R}\rangle\rangle + \langle\langle\mathbf{J}^{\text{EC}}_{R\to L}\rangle\rangle}{\langle\langle\mathbf{J}^R_{0\to 1} + \mathbf{J}^R_{1\to 0}\rangle\rangle\langle\langle\mathbf{J}^L_{0\to 1} + \mathbf{J}^L_{1\to 0}\rangle\rangle}\delta(\tau) \approx 1 + \frac{\langle\langle\mathbf{J}^{\text{CAR}}_{\text{in}}\rangle\rangle + \langle\langle\mathbf{J}^{\text{CAR}}_{\text{out}}\rangle\rangle + \langle\langle\mathbf{J}^{\text{EC}}_{L\to R}\rangle\rangle + \langle\langle\mathbf{J}^{\text{EC}}_{R\to L}\rangle\rangle}{\langle\langle\mathbf{J}^R_{0\to 1} + \mathbf{J}^R_{1\to 0}\rangle\rangle\langle\langle\mathbf{J}^L_{0\to 1} + \mathbf{J}^L_{1\to 0}\rangle\rangle}\delta(\tau), \end{aligned} \tag{8}$$

where we in the last step have omitted the weak correlations between separate processes taking place on different islands. For the instantaneous correlations, the timing jitter of the detectors plays an important role, leading to a broadening of the delta peak. To account for the timing jitter, we convolve the correlation function with a Gaussian distribution of width σ_D, which is computed from a weighted average of the timing noise of the different processes

$$\begin{aligned}\sigma_D &= \frac{\langle\langle \mathbf{J}_{\text{in}}^{\text{CAR}}\rangle\rangle\, \sigma_{\text{in}}^{\text{CAR}} + \langle\langle \mathbf{J}_{R\to L}^{\text{EC}}\rangle\rangle\, \sigma_{R\to L}^{\text{EC}} + \langle\langle \mathbf{J}_{L\to R}^{\text{EC}}\rangle\rangle\, \sigma_{L\to R}^{\text{EC}} + \langle\langle \mathbf{J}_{\text{out}}^{\text{CAR}}\rangle\rangle\, \sigma_{\text{out}}^{\text{CAR}}}{\langle\langle \mathbf{J}_{\text{in}}^{\text{CAR}}\rangle\rangle + \langle\langle \mathbf{J}_{R\to L}^{\text{EC}}\rangle\rangle + \langle\langle \mathbf{J}_{L\to R}^{\text{EC}}\rangle\rangle + \langle\langle \mathbf{J}_{\text{out}}^{\text{CAR}}\rangle\rangle} \\ &= 460\ \mu\text{s},\end{aligned} \tag{9}$$

where $\sigma_{\text{in}}^{\text{CAR}} = \sqrt{\sigma_{\tau_\text{j}(1\to0)}^{L}{}^2 + \sigma_{\tau_\text{j}(1\to0)}^{R}{}^2}$, $\sigma_{R\to L}^{\text{EC}} = \sqrt{\sigma_{\tau_\text{j}(0\to1)}^{L}{}^2 + \sigma_{\tau_\text{j}(1\to0)}^{R}{}^2}$, $\sigma_{L\to R}^{\text{EC}} = \sqrt{\sigma_{\tau_\text{j}(1\to0)}^{L}{}^2 + \sigma_{\tau_\text{j}(0\to1)}^{R}{}^2}$ and $\sigma_{\text{out}}^{\text{CAR}} = \sqrt{\sigma_{\tau_\text{j}(0\to1)}^{L}{}^2 + \sigma_{\tau_\text{j}(0\to1)}^{R}{}^2}$ are the sums of the relevant jitter noises for each process. The superscripts L and R on the timing jitters $\sigma_{\tau_\text{j}(i\to f)}$ here denote the left and right detector respectively. We then obtain the final expression

$$g_x^{(2)}(\tau) = 1 + \frac{\alpha_2}{\sqrt{2\pi}\sigma_D} e^{-\tau^2/(2\sigma_D^2)}, \tag{10}$$

with

$$\alpha_2 \equiv \frac{\langle\langle \mathbf{J}_{\text{in}}^{\text{CAR}}\rangle\rangle + \langle\langle \mathbf{J}_{\text{out}}^{\text{CAR}}\rangle\rangle + \langle\langle \mathbf{J}_{L\to R}^{\text{EC}}\rangle\rangle + \langle\langle \mathbf{J}_{R\to L}^{\text{EC}}\rangle\rangle}{\langle\langle \mathbf{J}_{0\to1}^{R} + \mathbf{J}_{1\to0}^{R}\rangle\rangle\langle\langle \mathbf{J}_{0\to1}^{L} + \mathbf{J}_{1\to0}^{L}\rangle\rangle} = 210\ \text{ms}, \tag{11}$$

which is Equation (2) of the main text. Here we see that the crossed Andreev reflections, together with elastic cotunneling, determine the integral over the distribution.

B. Waiting time distributions

We define the distribution of waiting times between two types of transitions α and β as [3]

$$\mathcal{W}(\tau|t) \equiv \tilde{P}_{\beta\alpha}(t+\tau|t), \tag{12}$$

where $\tilde{P}_{\beta\alpha}(t+\tau|t)$ is the *exclusive* probability density of observing a transition β at time $t+\tau$ provided that a transition α occurred at time t, with no other transition β taking place in between. For a steady state, the waiting time distribution depends only on the time difference, i.e., $\mathcal{W}(\tau|t) \equiv \mathcal{W}(\tau)$. In that case, we may express Equation (13) in terms of jump operators as

$$\mathcal{W}(\tau) = \frac{\langle\langle \mathbf{J}_\beta e^{(\mathbf{L}-\mathbf{J}_\beta)\tau}(\mathbf{J}_\alpha - \mathbf{J}_{\alpha\beta})\rangle\rangle}{\langle\langle \mathbf{J}_\alpha\rangle\rangle} + \eta_0\delta(\tau), \tag{13}$$

with $\eta_0 \equiv \langle\langle \mathbf{J}_{\alpha\beta}\rangle\rangle/\langle\langle \mathbf{J}_\alpha\rangle\rangle$, which includes the instantaneous correlations of two-electron processes involving both α and β transitions. Similar to the $g^{(2)}$-function, the instantaneous correlations are sensitive to the timing jitter of the detectors, as discussed for the cross-waiting time distribution below. The waiting time distribution is normalized, $\int_0^\infty \mathcal{W}(\tau)d\tau = 1$, and has the units of inverse time. Here, the delta function is defined such that $\int_0^\infty d\tau\delta(\tau) = 1$.

1. Equation (3) in the main text

We consider the distribution of waiting times between subsequent electrons tunneling out of the right island into the superconductor. To obtain this auto-waiting time distribution of the right island, we set $\mathbf{J}_\alpha = \mathbf{J}_\beta = \mathbf{J}_{1\to0}^{R}$ and $\mathbf{J}_{\alpha\beta} = 0$ in Equation (13), yielding the expression

$$\mathcal{W}(\tau) = \frac{\langle\langle \mathbf{J}_{1\to0}^{R} e^{(\mathbf{L}-\mathbf{J}_{1\to0}^{R})\tau}\mathbf{J}_{1\to0}^{R}\rangle\rangle}{\langle\langle \mathbf{J}_{1\to0}^{R}\rangle\rangle}. \tag{14}$$

Similar to the auto-correlation $g^{(2)}$-function, we may use the fact that the two-electron processes involving also the left island are slow compared to the right island, and that the latter may therefore be treated as an effectively separate

four-level system, whose dynamics is governed by the Liouvillian $\mathbf{L}_R$ in Equation (5). Treating the high-energy charge states -1 and $+2$ as short-lived virtual states, we find

$$\mathcal{W}(\tau) = \frac{1}{\langle\tau\rangle u}\left(e^{-\gamma(1-u)\tau/2} - e^{-\gamma(1+u)\tau/2}\right), \tag{15}$$

which is Eq. (3) in the main text. Here γ is the inverse correlation time in Equation (7), $\langle\tau\rangle = 1.2$ s is the average waiting time between two subsequent $1 \to 0$ transitions on the right island, and $u = \sqrt{1 - 4/(\gamma\langle\tau\rangle)}$. Numerical calculations show that this approximation agrees very well with what we obtain by numerically evaluating Equation (14).

2. Equation (4) in the main text

Last, we consider the distribution of waiting times between electrons tunneling out of the left island and electrons tunneling out of the right island. To obtain this cross-waiting time distribution, we set $\mathbf{J}_\alpha = \mathbf{J}^L_{1\to 0}$, $\mathbf{J}_\beta = \mathbf{J}^R_{1\to 0}$ and $\mathbf{J}_{\alpha\beta} = \mathbf{J}^{\mathrm{CAR}}_{\mathrm{in}}$ in Equation (13), yielding

$$\mathcal{W}_x(\tau) = \frac{\langle\!\langle \mathbf{J}^R_{1\to 0} e^{(\mathbf{L}-\mathbf{J}^R_{1\to 0})\tau}(\mathbf{J}^L_{1\to 0} - \mathbf{J}^{\mathrm{CAR}}_{\mathrm{in}})\rangle\!\rangle}{\langle\!\langle \mathbf{J}^L_{1\to 0}\rangle\!\rangle} + \eta_0\delta(\tau) \equiv (1-\eta_0)\mathcal{W}_0(\tau) + \eta_0\delta(\tau), \tag{16}$$

where $\eta_0 = \langle\!\langle \mathbf{J}^{\mathrm{CAR}}_{\mathrm{in}}\rangle\!\rangle / \langle\!\langle \mathbf{J}^L_{1\to 0}\rangle\!\rangle = 0.36$ and $\mathbf{J}^{\mathrm{CAR}}_{\mathrm{in}}$ is the superoperator of Cooper pair formation. A simple analytic expression for the first term, $\mathcal{W}_0(\tau)$, is challenging to obtain, however, it decays approximately as $\exp[-\gamma(1-u)\tau/2]$ for long waiting times. Similar to the cross-correlation $g^{(2)}$-function, the instantaneous correlations in the second term are broadened by the timing jitter of the detectors, resulting in a Gaussian distribution of width $\sigma_D = \sqrt{(\sigma^L_{\tau_{\mathrm{j}}(1\to 0)})^2 + (\sigma^R_{\tau_{\mathrm{j}}(1\to 0)})^2} = 410$ μs, here corresponding to the detector timing jitter of the $1 \to 0$ transitions only. However, in contrast to the $g^{(2)}$-function, the waiting time distribution is sensitive to the time ordering of the events. In half of the Cooper pair formation processes, the timing jitter will cause the transition $1 \to 0$ on the right island to be detected *before* the transition on the left. In those cases, the observed waiting time will extend until the next tunneling event, which to a good approximation is described by the auto-waiting time distribution in Equation (15). In conclusion, we thus find

$$\mathcal{W}_x(\tau) = (1-\eta_0)\mathcal{W}_0(\tau) + \frac{\eta_0}{2}\left[\sqrt{\frac{2}{\pi}}\frac{1}{\sigma_D}\exp[-\tau^2/(2\sigma_D^2)] + \mathcal{W}(\tau)\right], \tag{17}$$

which is Equation (4) in the main text. Just as for the cross-correlations, $g^{(2)}_x$, we see that the crossed Andreev reflections are important for the waiting time distribution, in particular, by giving rise to a large peak at short times.

[1] V. F. Maisi, O.-P. Saira, Y. A. Pashkin, J. S. Tsai, D. V. Averin, and J. P. Pekola, Real-Time Observation of Discrete Andreev Tunneling Events, Phys. Rev. Lett. **106**, 217003 (2011).
[2] W. Maichen, *Digital Timing Measurements. From Scopes and Probes to Timing and Jitter*, Frontiers in Electronic Testing: 33 (Springer US, 2006).
[3] H. J. Carmichael, S. Singh, R. Vyas, and P. R. Rice, Photoelectron waiting times and atomic state reduction in resonance fluorescence, Phys. Rev. A **39**, 1200 (1989).
[4] C. Emary, C. Pöltl, A. Carmele, J. Kabuss, A. Knorr, and T. Brandes, Bunching and antibunching in electronic transport, Phys. Rev. B **85**, 165417 (2012).
